# Incorporation of Dosimetric Gradients and Parotid Gland Migration Into Xerostomia Prediction

**DOI:** 10.3389/fonc.2019.00697

**Published:** 2019-07-31

**Authors:** Rosario Astaburuaga, Hubert S. Gabryś, Beatriz Sánchez-Nieto, Ralf O. Floca, Sebastian Klüter, Kai Schubert, Henrik Hauswald, Mark Bangert

**Affiliations:** ^1^Department of Medical Physics in Radiation Oncology, Deutsches Krebsforschungszentrum, Heidelberg, Germany; ^2^Medical Faculty of Heidelberg, Universität Heidelberg, Heidelberg, Germany; ^3^Heidelberg Institute for Radiation Oncology, Heidelberg, Germany; ^4^Institute of Physics, Pontificia Universidad Católica de Chile, Santiago, Chile; ^5^Medical Image Computing, Deutsches Krebsforschungszentrum, Heidelberg, Germany; ^6^Department of Radiation Oncology, University Hospital Heidelberg, Heidelberg, Germany; ^7^Clinical Cooperation Unit Radiation Oncology, Deutsches Krebsforschungszentrum, Heidelberg, Germany

**Keywords:** head and neck cancer, xerostomia, intensity-modulated radiotherapy, daily MVCT, anatomical changes, dosimetric changes, normal tissue-complication probability

## Abstract

**Purpose:** Due to the sharp gradients of intensity-modulated radiotherapy (IMRT) dose distributions, treatment uncertainties may induce substantial deviations from the planned dose during irradiation. Here, we investigate if the planned mean dose to parotid glands in combination with the dose gradient and information about anatomical changes during the treatment improves xerostomia prediction in head and neck cancer patients.

**Materials and methods:** Eighty eight patients were retrospectively analyzed. Three features of the contralateral parotid gland were studied in terms of their association with the outcome, i.e., grade ≥ 2 (G2) xerostomia between 6 months and 2 years after radiotherapy (RT): planned mean dose (MD), average lateral dose gradient (GRADX), and parotid gland migration toward medial (PGM). PGM was estimated using daily megavoltage computed tomography (MVCT) images. Three logistic regression models where analyzed: based on (1) MD only, (2) MD and GRADX, and (3) MD, GRADX, and PGM. Additionally, the cohort was stratified based on the median value of GRADX, and a univariate analysis was performed to study the association of the MD with the outcome for patients in low- and high-GRADX domains.

**Results:** The planned MD failed to recognize G2 xerostomia patients (AUC = 0.57). By adding the information of GRADX (second model), the model performance increased to AUC = 0.72. The addition of PGM (third model) led to further improvement in the recognition of the outcome (AUC = 0.79). Remarkably, xerostomia patients in the low-GRADX domain were successfully identified (AUC = 0.88) by the MD alone.

**Conclusions:** Our results indicate that GRADX and PGM, which together serve as a proxy of dosimetric changes, provide valuable information for xerostomia prediction.

## 1. Introduction

The advent of machine learning methods is changing normal tissue complication probability (NCTP) modeling in radiotherapy (RT). The common standard of univariate models relying on simple dosimetric descriptors (1) is more and more challenged by complex inference algorithms taking into account a multitude of factors, ranging from patient demographics to complex radiomic and dosiomic features (2–6). At the same time, image-guided radiotherapy (IGRT) provides more and better daily images of the treated anatomy, increasing the precision and accuracy of radiation delivery (7). Due to the sharp dose gradients of IMRT dose distributions, even subtle anatomical changes may lead to differences between the planned and the delivered dose distributions—especially within the high gradient regions around the target edges. Yet still, current NTCP studies are still largely based on the planned dose (8–12) and not on the actually delivered dose causing the radiation effect. Patient-specific dose reconstruction involving daily imaging, dose calculation, deformable image registration, and dose accumulation remains very challenging (13, 14).

This study addresses the possible impact of anatomical changes on radiation-induced toxicity. Therefore, we focus on xerostomia (dryness of mouth) for head-and-neck (HN) cancer patients. This side effect is usually related to the parotid glands which are responsible for more than 50% of the total salivary volume (15–17).

Standard xerostomia prediction models are based on the parotid gland's mean dose (18–21). The Quantitative Analyses of Normal Tissue Effects in the Clinic (QUANTEC) group recommends sparing at least one parotid gland to a mean dose < 20 Gy or both parotid glands to a mean dose < 25 Gy in order to prevent severe xerostomia (22). However, recent studies for highly conformal intensity-modulated radiation therapy (IMRT) treatments question the reliability of parotid gland mean dose models (2, 23). A study of our group, in which we retrospectively analyzed a cohort of 153 patients treated at Heidelberg University Hospital (23), aimed to investigate whether standard NTCP models based on parotid gland mean dose are suitable for the prediction of xerostomia in modern IMRT treatments. We found that the planned mean dose failed to predict G2 xerostomia patients, highlighting the need of new predictive models in this domain. Instead of the mean dose, we then observed a significantly higher predictive power of the parotid gland's average lateral dose gradient and volume (4) in the same cohort of patients. For prostate patients, the dose gradient was found to code for the amount of dosimetric deviation from the planned dose during treatment in the presence of anatomical changes (24). The neglected anatomical motion may also be a viable explanation for the good performance of the dose gradient in our cohort: for HN cancer patients parotid gland migration predominantly occurs toward the medial direction during treatment (25–30) and consequently leads to an increase of the parotid gland dose compared to planning (31).

Together, these findings suggest that anatomical changes may blur the relationship between an increased planned mean dose and xerostomia. The lateral dose gradient may act as a proxy of a potential dose increment within the parotid gland due to the motion of the parotid gland toward medial during the treatment (25–30).

This paper investigates the added value of patient-specific information about the average lateral dose gradient within the parotid gland and about the amount of parotid gland migration toward the medial direction for xerostomia prediction in a subgroup of the cohort analyzed in Gabryś et al. (4, 23). We propose and test two new models for G2 xerostomia prediction in the modern IMRT era. The first model considers the planned mean dose and the planned average dose gradient in the lateral direction, as an indirect way to account for potential dose increment within the parotid gland in case of inter-fractional motion toward medial. The second model directly includes the estimated parotid gland migration during treatment. Therefore, we compare the performance of the standard univariate mean dose model for xerostomia prediction to the performance of multivariate models based on the mean dose, the average lateral dose gradient, and the parotid gland migration. We present an intuitive interpretation of the results and discuss potential issues within our data set.

## 2. Materials and Methods

### 2.1. Patients

Eighty eight HN cancer patients treated with IMRT at the Heidelberg University Hospital between 2008 and 2011, using daily MVCT imaging-based setup, were retrospectively analyzed. The cohort corresponds to a subset of patients from a previously published study (4, 23) for whom appropriate daily MVCT imaging data was available. Patient and tumor characteristics are listed in Table 1. The average mean dose in the parotid glands was rather low (25.6 ± 8.9 Gy and 18.6 ± 5.9 Gy for ipsi- and contra-lateral parotid glands, respectively), indicating a high degree of dose conformality within our cohort. The study was approved by the Ethics Committee of the Medical Faculty of Heidelberg University.

**Table 1:** Patient and tumor characteristics for the complete cohort, stratified by negative and positive cases.

	**All**	**G0–1 xer**.	**G2 xer**.
Total number of patients	88	78	10
**Age**			
Median	61	61	55
Q1–Q3	54–66	55–65	51–68
Range	39–82	39–82	43–74
**Sex**			
Female	21	19	2
Male	67	59	8
**Tumor site**			
Hypopharynx/Larynx	14	14	0
Nasopharynx	9	8	1
Oropharynx	62	54	8
Other	3	2	1
**Treatment modality**			
Conventional IMRT	3	2	1
Tomotherapy	85	76	9
**Ipsilateral parotid mean dose [Gy]**			
Median	24.5	24.4	26.1
Q1–Q3	20.7–26.9	20.5–26.8	21.8–27.2
Range	11.2–63.4	11.2–61.4	17.3–63.4
**Contralateral parotid mean dose [Gy]**			
Median	20.0	19.8	20.4
Q1–Q3	15.5–22.5	15.4–22.3	16.4–23.1
Range	4.1–30.9	4.1–30.9	15.1–26.1

*Q1 and Q3 correspond to the first and third quartiles, respectively*.

### 2.2. Endpoint

Three hundred and ninety five routine xerostomia toxicity follow-up reports between 6 months and 2 years after RT were analyzed. We aimed to predict moderate-to-severe xerostomia defined as grade 2 or higher (G2) according to the Common Terminology Criteria for Adverse Effects (CTCAE) (32). Two hundred and eighty five reports (72%) included quantitative information according to CTCAE^1^. In case only descriptive information was provided, the scores were consistently assigned in retrospect, according to a set of rules together with Heidelberg University Hospital clinicians. According to the time intervals defined by Gabryś et al. (4), late (6–15 months) and long-term (15–24 months) follow-up reports were pooled together in order to increase the patient number. In case there were multiple follow-up reports available for individual patients, the final toxicity score was calculated as the arithmetic mean rounded to the nearest integer with x.5 being rounded up. No patients with xerostomia grade higher than 2 were observed in this cohort. Therefore, positive and negative groups comprised grade 2 and grade 0 to 1 xerostomia patients, respectively.

### 2.3. Feature Inclusion and Extraction

We considered the association of xerostomia with three different features:

1. The parotid gland mean dose (MD) was included as a standard predictor for xerostomia (18, 19, 21, 22).
2. The average dose gradient within the parotid gland in the lateral direction (GRADX) was included as it has been previously established as a significant predictor for G2 xerostomia between 15 and 24 months after RT in the considered patient cohort (4).
3. The distance of parotid gland migration toward medial (PGM) over the course of the treatment was included to code for anatomical changes.

**Figure 1:**
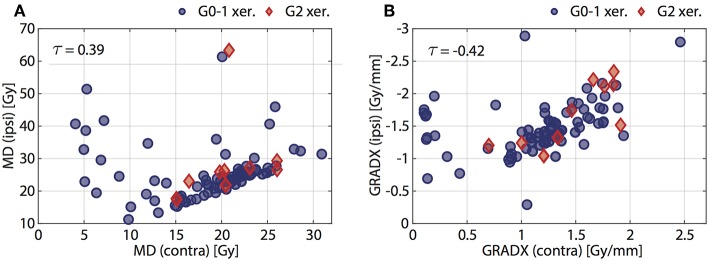
Scatter plots of **(A)** planned mean dose (MD), and **(B)** average dose gradients (GRADX) in ipsilateral gland vs. contralateral gland. The Kendall's τ is given for each feature. Positive (G2 xer.) and negative (G0-1 xer.) patients are indicated with red diamonds and blue circles, respectively.

Mathematical feature definitions for (1, 2) and technicalities regarding feature extraction are laid out in due detail in the appendix of Gabryś et al. (4). Corresponding data is shown in Figure 1. The Kendall rank correlation coefficient (τ) between identical features for the contra- and ipsilateral gland of each patient always exceeded 0.4, which corresponds to a 70% chance that a contra- and ipsilateral feature rank a random pair of observations in the same way. Consequently, we restricted further investigations to the contralateral gland. The following section provides an in-depth description of the computation of PGM.

### 2.4. Parotid Gland Migration

We estimated parotid gland migration through its linear correlation with the relative volume change of the external contour at the level of the C2 vertebral body, according to Barker et al. (25). Due to limited availability of daily MVCT image guidance data covering the level of interest exactly at the end of the treatment, we calculated the volume change between the first fraction and a control day (between 28 and 36 days after the first fraction). We argue that this is a meaningful approach as (1) changes in the external contour manifest usually in a significant way during the second half of treatment (25) and (2) the migration of both parotid glands is equal and approximately linear during treatment (26). In particular, the linearity of motion enables the extrapolation of PGM, i.e., the parotid gland migration at the end of the treatment, based on the parotid gland migration at control day *c* (PGM_*c*_).

**Figure 2:**
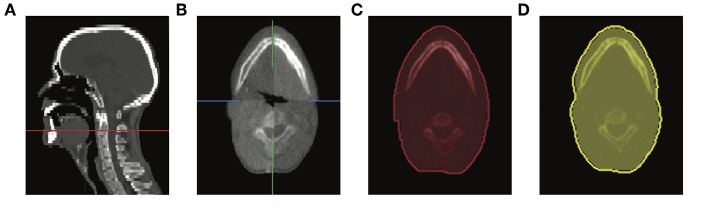
Screen captures of MITK showing **(A)** the level of the C2 vertebral body in sagittal view of the planning kVCT (red line), **(B)** evaluation of rigid registration between first (bottom-left and upper-right) and control day (bottom-right and upper-left) MVCTs, external contours at the level of interest corresponding to the **(C)** first fraction and **(D)** control day.

Daily (MVCT) and planning (kVCT) image data were analyzed with the Medical Imaging Interaction Toolkit MITK^2^ (33). First, MVCT images corresponding to the first fraction and the control day were automatically aligned to the kVCT images with the multimodal rigid translation (34). The kVCT image was considered as the reference because it offered better recognition of the C2 vertebral body due to higher image contrast and resolution. Next, the external contour at the level of the C2 vertebral body was segmented in both MVCT images. The external contours were created with an automated region growing tool and manually corrected where considered necessary after visual inspection. In this step we focused on excluding the fixation mask from the external contour, as the aim was to quantify the anatomical changes and not variations in the fixation mask. The identification of the axial region of interest was based on kVCT planning images, as they offer higher contrast and resolution. Therefore, we counted the vertebrae and selected the second cervical. We paid special attention to identifying the dens or odontoid process, the most pronounced feature of C2 vertebra. The relative volume change at the control day *c* was computed according to ΔVC2_c_ = (VC2_c_ − VC2_1_)/VC2_1_, where VC2_1_ and VC2_c_ are the volumes of the external contour at the level of interest extracted from the first and control day, respectively. Figure 2 illustrates the steps. Finally, the parotid gland migration toward medial between the first and control day was calculated in millimeters, according to the linear relationship^3^ (25)

(1) $$\begin{array}{l} \text{PG}{\text{M}}_{\text{c}}=-0.413\cdot \Delta \text{VC}2_{\text{c}} \end{array}$$

and linearly extrapolated in time (26) to obtain the amount of parotid gland migration at the end of the treatment, PGM.

### 2.5. Logistic Regression Models

Three logistic regression models of xerostomia were investigated. First, a model based on the mean dose to the contralateral parotid gland (MD). Second, a model based on the mean dose and the average dose gradient in the lateral direction within the contralateral parotid gland, including the interaction term (MD + GRADX + MD · GRADX). Third, the same as the second model but additionally with the parotid gland migration component (MD + GRADX + MD · GRADX + PGM).

The performance of each model was evaluated with the area under the receiver operating characteristic curve (AUC). The reported AUCs were estimated with leave-pair-out cross-validation (35). The confidence intervals (CI) for the AUC estimates were calculated with the bias-corrected and accelerated (BCa) bootstrap (36).

The model's behavior in terms of high and low complication probability regions of the input space was analyzed with partial dependence plots (37, 38). These plots present the average response of a model as the value of the analyzed variable changes.

### 2.6. Stratification of the Cohort

In order to investigate whether the predictive power of the MD depends on the extent of GRADX, the cohort was split into high- and low-GRADX groups. We used the median value of the GRADX within the contralateral gland to obtain two groups with the same number of cases.

A univariate analysis was performed to study the association of the MD with the outcome of the two groups. High MD was considered a risk factor for radiation-induced xerostomia (22). The predictive power was quantified with the AUC.

## 3. Results

### 3.1. Parotid Gland Migration

The median volume change (± one standard deviation) of the external contour at the level of the C2 vertebral body between the first fraction and the control day was Δ*VC*2_*c*_ = −2.0% ± 4.2%. Considering a linear movement of parotid glands over time (26), the median parotid gland migration in the medial direction at the end of the treatment was PGM = 1.03 ± 2.2 mm.

**Figure 3:**
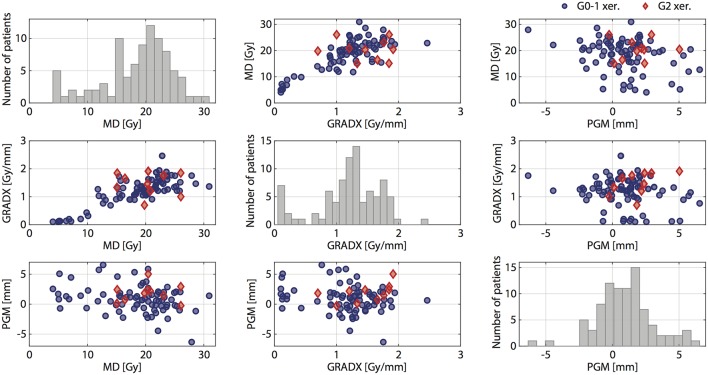
Pair plot for MD, GRADX, and PGM. Positive (G2 xer.) and negative (G0-1 xer.) patients are indicated with red diamonds and blue circles, respectively.

Figure 3 shows a pair plot for MD, GRADX, and PGM. We observed that more than half of the studied cohort (67%) experienced parotid gland movement toward medial during treatment. Among the non-G2 xerostomia patients (negative cases), 63% had migration of parotid glands toward medial. Among the G2 xerostomia patients (positive cases), 9 of 10 or 90% had parotid gland migration toward the medial.

### 3.2. Logistic Regression Models

In the univariate prediction model based on the mean dose only, we observed a positive dose dependence, i. e., the complication probability was higher for larger MD values. However, the planned MD alone failed to reliably recognize G2 xerostomia with a model performance of AUC = 0.57 (95% CI: 0.43–0.71). With this we reconfirmed that the planned MD alone also failed to recognize G2 xerostomia in a reduced sample of the cohort analyzed in Gabryś et al. (4, 23).

**Figure 4:**
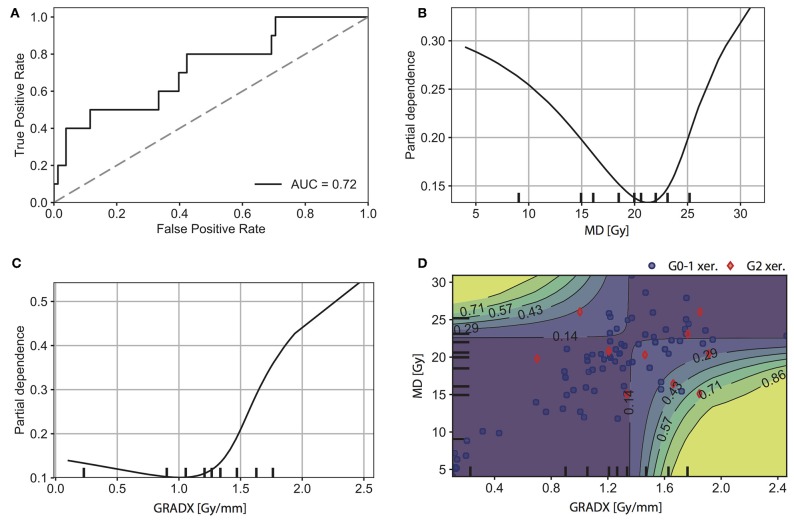
Results for the second model (MD + GRADX + MD· GRADX). **(A)** ROC curve. 1D partial dependence plot for **(B)** MD, and **(C)** GRADX. **(D)** 2D partial dependence plot for MD and GRADX. The rug plots (black marks along the axes) represent the distribution of the data and correspond to percentiles of observations. Intuitively, we were more confident in regions with higher density of observations. Positive (G2 xer.) and negative (G0-1 xer.) patients are indicated with red diamonds and blue circles, respectively.

Based on a multivariate model which additionally included GRADX and the associated interaction term MD · GRADX, we observed a model performance of AUC = 0.72 (95% CI: 0.57–0.85) (Figure 4A). The corresponding ROC curve and partial dependence plots are shown in Figure 4. Figure 4B shows a parabolic relation between MD and complication probability with a minimum at ~21 Gy. As illustrated in the partial dependence plot of Figure 4C, the complication probability was higher for larger values of GRADX. The parabolic behavior of the MD is further illustrated in the two-dimensional partial dependence plot of Figure 4D: for patients with GRADX values lower than 1.3 Gy/mm the complication probability increased with increasing MD. On the contrary, for patients with GRADX values higher than 1.3 Gy/mm, the complication probability decreased with increasing MD values.

**Figure 5:**
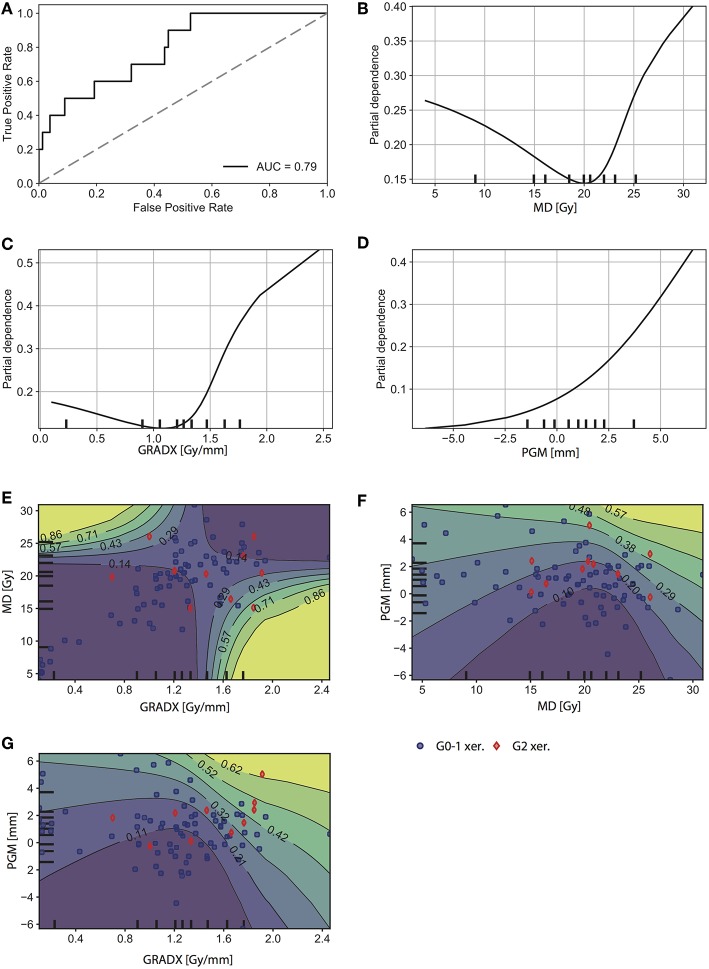
Results for the third model (MD + GRADX + PGM + MD· GRADX). **(A)** ROC curve. 1D partial dependence plot for **(B)** MD, **(C)** GRADX, and **(D)** PGM. 2D partial dependence plot for **(E)** MD and GRAX, **(F)** PGM and MD, and for **(G)** PGM and GRADX. The rug plots (black marks along the axes) represent the distribution of the data and correspond to percentiles of observations. Intuitively, we were more confident in regions with higher density of observations. Positive (G2 xer.) and negative (G0-1 xer.) patients are indicated with red diamonds and blue circles, respectively.

Finally, by also including information about the parotid gland movement during treatment, the model performance increased to AUC = 0.79 (95% CI: 0.67–0.89) (Figure 5A). For this model, we observed similar 1D partial dependence for MD, GRADX, and 2D partial dependence between MD and GRADX as in the previous model (Figures 5B,C,E). As expected, the complication probability was higher for larger values of PGM (Figure 5D). Similarly, Figures 5F,G show that the complication probability increases with increasing PGM for a fixed MD or GRADX value. For PGM higher than ~2 mm, GRADX only had a meaningful impact on the complication probability for values greater than ~1.3 Gy/mm (Figure 5G).

### 3.3. Stratification of the Cohort

The two-dimensional partial dependence plots shown in Figures 4D, 5E indicate that the MD seems to behave differently in high- and low-GRADX domains. To further investigate this, we stratified the cohort based on the median value of GRADX within the contralateral gland (GRADX = 1.27 Gy/mm), and evaluated the model performance of the MD in the two domains. For patients in the low-GRADX domain, the MD successfully predicted G2 xerostomia with an AUC = 0.85 (95% CI: 0.68–0.95) with the complication probability increasing with increasing MD. For patients in the high-GRADX domain, the MD failed to recognize positive patients AUC = 0.34 (95% CI: 0.15–0.55). Note that AUC < 0.5 indicates a directional flip of the risk factor. Therefore, the AUC = 0.34 for the risk factor of a higher MD is equivalent to the AUC = 1 - 0.34 = 0.66 (95% CI: 0.45–0.85) for the risk factor of a lower MD.

## 4. Discussion

Based on a linear correlation with the volume change of the external contour on the level of the C2 vertebral body (25), we observed that the majority of patients in our cohort experienced parotid gland migration toward the medial direction. As the analyzed patients were not re-planned during the course of the treatment, we must assume that the actually delivered parotid gland dose was higher than the planned dose for the majority of the patients. This observation highlights the potential importance of parotid gland migration toward the high dose region on the development of xerostomia.

Lee et al. (39) found that the planned MD alone was not sufficient for the prediction of xerostomia after tomotherapy. Similarly, in our cohort we found that the planned MD failed to recognize G2 xerostomia patients (AUC = 0.57). The reasons for this finding may be myriad. First, the dosage to parotid glands was lower in our cohort than in most of the studies which supported the mean dose model (21, 40–46). In fact, older studies on xerostomia were based on 3D conformal treatments and early IMRT methods, whereas our cohort was treated with modern IMRT (the majority with tomotherapy) allowing for highly conformal treatment plans with low doses to parotid glands. It is possible that the mechanisms of dose response in a low-dose domain may be better explained by different dose descriptors. Second, the dose gradients resulting from high conformality of the dose distribution made the planned dose more susceptible to variation coming from treatment uncertainties. This may increase the discrepancy between the planned mean dose and the actually delivered dose (and therewith the side effect). Third, the clinical goal of meeting the QUANTEC recommendations (22) imposes an homogenization in the mean dose values during treatment planning. Simply put, if we generate all treatment plans to have a mean dose below 25 Gy, then the variation in mean dose is reduced. This introduces a bias in the cohort and reduces the information content of the mean dose for xerostomia modeling.

We found that the inclusion of GRADX allows us to improve the prediction of radiation-induced G2 xerostomia. We suggest that the average dose gradient in the lateral direction within the parotid gland might be an indicator of the increment in delivered mean dose when parotid gland migration toward medial is present. This hypothesis is supported by similar work by Jiang et al. for the prostate, where the authors investigated the impact of internal organ motion on IMRT treatment planning. They found that the amount of percentage mean dose deviation depends on the dose gradient and organ motion (24).

Within our study, we consistently observed increasing xerostomia risk with increasing GRADX and PGM. This result supports the intuitive interpretation of potential anatomical changes yielding a more pronounced increase in the parotid gland dose for patients with a high lateral dose gradient in the parotid gland and/or suffering from strong parotid gland migration toward the medial direction. For MD, however, the interaction models and the stratification exposed that a reduced mean dose is a risk factor within our cohort for patients with a high lateral dose gradient within the parotid glands. This should be interpreted with care, as the corresponding CI for the stratified cohort includes AUC = 0.5 which questions the significance of the observation. Further, our data set featured a number of patients with particularly low parotid gland mean doses. It is debatable to what extent the standard mean dose model which has been motivated for patient cohorts with substantially higher mean doses (traditionally for 3D conformal RT) is applicable in this low dose domain (23).Visual inspection of the pair plots in Figure 3 shows large overlap between G2 and non-G2 xerostomia patients. However, quantitative analysis of MD+GRADX showed that even with such overlap, the model based on these two parameters provided reasonably good predictions (AUC = 0.72).

The aim of this study was not to provide a final xerostomia prediction model that can be used in the clinic, but rather to investigate the added value of including information on the dose gradient in the main direction of parotid gland movement during head and neck radiotherapy, as a surrogate of a potential mean dose increment.Yet still, some aspects of our study may help to directly inform clinical practice. As MD and GRADX are readily available from the treatment planning systems, these quantities could help identify patients where special care should be taken. For patients with a combination of MD and GRADX above a certain threshold, it could be advisable to monitor daily weight-loss and, if possible, parotid gland migration or shrinkage via daily imaging more closely in order to decide about re-planning. Simpler non-dosimetric descriptors, such as the patient's inter-fractional weight changes [that has been correlated with parotid gland migration (25) and shrinkage (47, 48)], could potentially improve the prediction of xerostomia.

Anatomical changes in the head and neck region may include not only parotid gland migration (i.e., a rigid movement of the parotid), but also parotid gland shrinkage (i.e., a volume change of the parotid itself). However, due to limitations in our underlying imaging data, we could not explicitly model parotid gland shrinkage; our study focuses on parotid gland migration which was quantified through its linear correlation with the reduction of the external contour at the level of C2 vertebral body (25). Therefore, parotid gland migration corresponded to a positional change of the parotids' center of mass (COM). However, changes in the parotid's COM might also occur due to parotid gland shrinkage, as it has been observed that shrinkage occurred as a contraction of the lateral aspects of the parotid glands (49). Consequently, there is only a poor differentiation between these two anatomical changes and their dosimetric consequences. Nevertheless, a rigid COM translation may also act as a proxy for dosimetric changes induced by PG shrinkage. We understand this approach as a first order approximation in modeling the full complexity of anatomical changes happening during treatment.

Furthermore, our data did not allow for a full dosimetric reconstruction of the actually delivered dose. As the MVCTs from daily positioning only have a limited field of view, it was impossible to accumulate dose for the entire parotid gland. Consequently, we considered the amount of parotid gland migration toward the medial direction as a proxy of dosimetric change during treatment. Obviously, this approach neglects a number of phenomena: (a) shifts of the parotid do not only take place toward medial (mean 3.4 mm) but also toward posterior and superior (means 2.7 mm and 1 mm) (29). (b) The parotid glands deform and shrink during treatment up to 30% (50–52). (c) The anatomical changes induce complex non-linear dosimetric changes that can only be accurately modeled with a full volumetric dose calculation. (d) The facilitated linear correlation model between the volume change of the external contour at the level of the C2 vertebral body and parotid gland migration itself makes wide approximations. In the future, it would be desirable to extend our model to also include volume deformations and changes in combination with dose recalculation and accumulation.

Another limitation of our study concerns the low number of patients included (88), from which only 11.4% presented complications. However, due to highly conformal treatments indicated by low average mean doses to the parotid glands (25.6 ± 8.9 Gy and 18.6 ± 5.9 Gy for ipsi- and contra-lateral parotid glands, respectively), such reduced xerostomia incidence was expected (39, 53). This limitation is transparent in the pair plot of Figure 3 and restricts model comparison with statistical tests. Nevertheless, even though low size of the minority class reduced the statistical power of the analysis, the presented results are statistically significant.

Nevertheless, we see an improvement in the prediction of G2 xerostomia by including the GRADX and the estimated PGM, as well as the associated interaction terms. Both factors are strong indicators for the deviation of the delivered dose from the planned dose. Consequently, our study motivates further research along the lines of image-guided normal tissue complication probability models that make use of sound information about anatomical changes happening during radiotherapy. By correlating outcome with the actually delivered dose and not with the planned dose, it may be possible to make even more reliable predictions of radiation therapy side effects in the future.

## 5. Conclusions

We have investigated xerostomia prediction in the context of anatomical changes occurring during the course of radiation treatments. For a cohort of 88 head and neck cancer patients, we found that 67% of the patients experienced parotid gland migration toward the medial direction (median 1.0 mm). In this cohort, a conventional xerostomia prediction model based on the parotid gland mean dose alone failed to reliably recognize xerostomia (AUC = 0.57). The second prediction model, based on the mean dose and the lateral dose gradient of the parotid gland, yielded improved predictive performance (AUC = 0.72). The third prediction model, based on the mean dose, the lateral dose gradient, and the amount of parotid gland migration toward the medial direction, yielded the highest AUC of 0.79. Our results indicate that the average lateral dose gradient and the parotid gland migration, which together serve as a proxy of dosimetric changes, provide valuable information for xerostomia prediction.

## Data Availability

The datasets for this study will not be made publicly available because the ethic statement does not allow to publish the dataset.

## Ethics Statement

This study was conducted in accordance with the Declaration of Helsinki and was approved by the Ethics Committee of Heidelberg University. Nr. S-392/2016 Validation and development of probabilistic prediction models for radiation-induced xerostomia. Written informed consent has been obtained from all study participants.

## Author Contributions

RA, HG, SK, KS, HH, and MB contributed to the acquisition of the clinical data. RA, HG, BS-N, and MB contributed to the analysis of the follow-up data. RF contributed to the analysis of MVCT and kVCT images. RA, HG, BS-N, and MB contributed to the methodology. RA and HG performed feature extraction and data visualization. RA performed the estimation of parotid gland migration. HG performed the statistical analysis. RA, MB, and HG drafted the manuscript. MB was the senior author supervising the project.

### Conflict of Interest Statement

The authors declare that the research was conducted in the absence of any commercial or financial relationships that could be construed as a potential conflict of interest.

## Footnotes

^1^Either CTCAE v3.0 or v4.03 grading scale was available in the follow up reports. As xerostomia definitions are identical in both versions, no inconsistency in grading was introduced.

^2^www.mitk.org

^3^The exact correlation was extracted from Figures 3 and 8 of Barker et al. (25), using a plot digitizer.

## Abbreviations

MD: mean value of the radiation dose to the parotid gland
GRADX: average value of the radiation dose gradient in the lateral direction within the parotid gland
PGM: distance of parotid gland migration toward medial over the course of the radiotherapy treatment.
